# Design and bio-inspired optimization of direct contact membrane distillation for desalination based on constructal law

**DOI:** 10.1038/s41598-020-73964-7

**Published:** 2020-10-08

**Authors:** Amir H. Keshavarzzadeh

**Affiliations:** School of Mechanical Engineering, University of Tehran, P.O. Box 11155-4563, Tehran, Iran

**Keywords:** Devices for energy harvesting, Applied physics

## Abstract

At the present study, a one-dimensional model for the flat sheet direct contact membrane distillation (DCMD) for desalination purposes is proposed. Flows and membrane properties have been estimated by appropriate temperature-dependent correlations. Results show that the numerical model is in a very good agreement with experimental data at various feed temperatures, flow rates and concentrations. A constructal design is investigated for DCMD to assess how constructal law can improve the DCMD performance. With the same thermal efficiency of 93.5%, constructal design improves the water mass flux by 37.5% in comparison with the conventional DCMD design. Also, an evolutionary-based optimization algorithm is employed to increase the efficiency of the constructal and conventional design. The Pareto frontier of the constructal and conventional design is compared with each other and the correlations between design variables are investigated. Overall, the present study demonstrates how constructal law can increase the performance of energy systems with a simple modification.

## Introduction

The deficiency of freshwater resources would cause a serious crisis in the foreseeable future. Searching for an efficient desalination method, as a result, has been surged in the past recent years. Among conventional desalination methods, membrane distillation (MD) is one of the newest technologies that is under development^1–5^. MD is a thermally driven system which water vapor is condensed by crossing the hydrophobic membrane. This technology could be used in a vast range of applications ranging from food industries to wastewater treatments. This technology benefits from several advantages. Operating in a lower temperature in comparison with other desalination systems such as multiple-effect desalination (MED) system and the ability to operate in highly saline water are two of the major advantages of this technology^6–10^. Such merits make the MD suitable for integrating with solar flat plate collectors. Several MDs are investigated such as direct contact membrane desalination (DCMD), vacuum membrane desalination (VMD), air gap membrane desalination (AGMD) and swiping gas membrane desalination (SGMD). At the present study, DCMD has been chosen with a focus on the flat sheet membrane configuration^11^. Countless mathematical models were developed to simulate the DCMD performance^12–14^.

Bandini et al.^15^ studied the effect of different operating conditions and membrane properties on the efficiency and mass transfer of the DCMD. Their results show that applying polarization coefficient alone, cannot lead to a satisfactory analysis of various resistances. They suggested novel sensitivity factors for the distillate flux based on convective heat transfer rate, membrane heat and mass transfer coefficient.

Al-Obaidani et al.^13^ conducted an experiment, investigating the hollow fiber DCMD exergy analysis, sensitivity study and economic evaluation in an effort to provide an optimization guideline. Hwang et al.^16^ carried out an experimental and analytical study on the flat sheet DCMD. They studied the impact of module dimensions and operating parameters on the DCMD performance. They also, obtained mass transfer coefficients for different module designs.

Bahmanyar et al.^17^ studied the impact of membrane morphology and operating parameters on the DCMD performance. The effect of those parameters on the concentration polarization and temperature was examined. Their results demonstrated that one of the most prominent parameters for permeate flux is the feed inlet temperature. Also, they showed that the feed temperature and feed circulation velocity have a meaningful correlation with concentration polarization.

Wu et al.^18^ investigated the impact of the membrane thickness on the transmembrane flux for DCMD. They calculated that the optimal membrane thickness increases with the reduction of the heat transfer coefficient and feed inlet temperature. However, the optimal membrane thickness decreases with the drop of the feed salinity and membrane permeability.

Bouchrit et al.^19^ evaluated the capability of treating hypersaline solution using flat sheet DCMD. They developed a model for mass and heat transfer and studied the effect of operating parameters on the DCMD performance. They determined the optimum operation parameters and constructed a lab-scale DCMD to concentrate the RO discharge for achieving the super-saturation saline with the aim of the salt crystallization. A long-term experiment was also constructed to monitor the permeability and the scaling of the membrane.

Imdakm and Matsuura^20^ developed a Monte Carlo simulation model to study the effect of membrane physical properties such as membrane pore size distribution, thickness, porosity, and thermal conductivity on the membrane flux. They proposed a DCMD’s porous membrane by a three-dimensional network model of the interconnected cylindrical pores with the distributive effective pore sizes.

Deshpande et al.^21^ completed a systematic numerical analysis, focusing on exergy destruction inside the hollow fiber DCMD module. They investigated the impact of different design and operation parameters on the exergy efficiency and recovery ratio of the DCMD system. They found that regardless of packing arrangement, the higher inlet feed temperature, the better recovery ratio. Also, they pointed out the important role of membrane properties on the recovery ratio.

Heat transfer is of great importance in a vast range of technologies. The very epitome of this could be seen at the heat exchangers, fuel cells and so forth. DCMD is one of the technologies which is highly dependent on the heat transfer. Multi-objective optimization, therefore, has been employed to obtain the optimized operation conditions^22–26^. Bejan et al.^27,28^ calculated the suitable size and shape of the heat exchanger which leads to a minimum thermodynamic loss. Bejan’s theory, constructal law, has the ability to increase the rate of the heat transfer significantly. In broad terms, constructal law can enhance the effectiveness of heat exchangers^29^. For instance, constructal theory can reduce the system cost by 50% in comparison with its conventional system^30^.

The present study tries to improve the performance of the flat sheet DCMD by combining the DCMD enhanced model with constructal law. The Nusselt correlation which considers the entry effects (both thermal and hydrodynamic) for rectangular cross-sections^31^ is also employed to improve the accuracy of the model. The most commonly cited issues in the literature were lack of proposing a constructal design with a thorough analysis of the DCMD design parameters. The following highlight the novelty of this research and its importance:

- Proposing a novel constructal DCMD design.
- Conducting a thorough analysis of DCMD design and operational parameters.
- Conducting an evolutionary-based optimization for constructal and conventional DCMD design.
- The performance of constructal and conventional DCMD design is compared with each other.
- Correlations between decision parameters are obtained.

### Modeling

**Figure 1:**
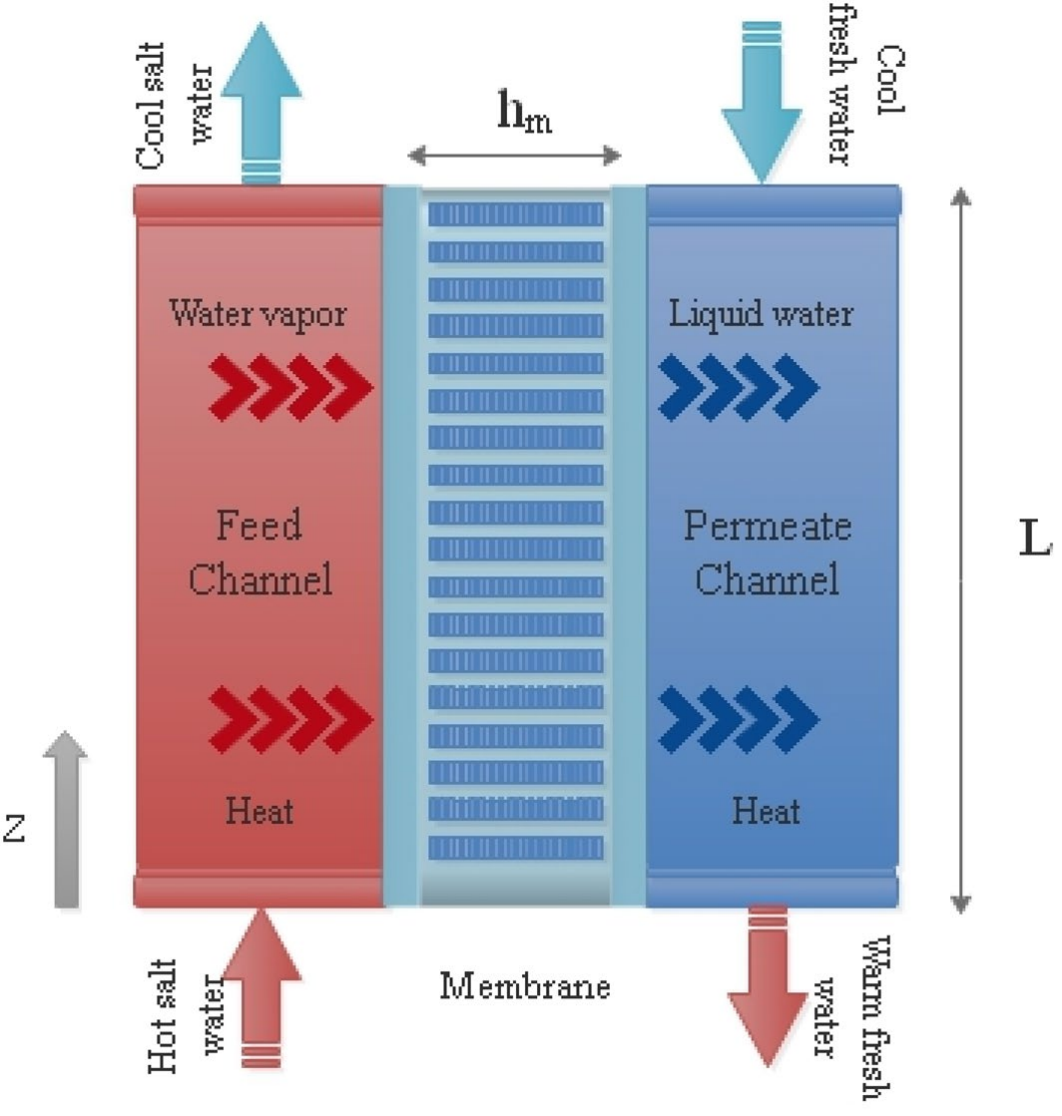
The schematic of the DCMD module.

The schematic of the flat sheet DCMD is illustrated in Fig. 1. The DCMD consists of three main parts namely: feed channel, permeate channel and membrane. The whole flat plate is divided into smaller control volumes and governing equations are solved for each control volume. It is worth mentioning that because of the coupling nature of governing equations these control volumes should be co-directed along the width of the DCMD.

While hot saltwater flows through the feed channel, cold freshwater streams in the opposite direction at the permeate channel whereas a porous hydrophobic membrane separates these water streams. This temperature difference causes a water vapor pressure gradient which is perpendicular to the membrane direction. The mass transfer through the membrane is proportional with the pressure gradient^32^:

1 $$ J_{m} = B\left( {P_{w.f.m} - P_{w.p.m} } \right) $$

where $P_{w.f.m}$ and $P_{w.p.m}$ are the water pressure at the feed and permeate channel, respectively. B is the membrane flux coefficient which can be evaluated by empirical or theoretical correlations. Chung et al.^33^ proposed an empirical equation to evaluate the mass flux through the membrane. Although the membrane flux coefficient changes with operating conditions, some of existed DCMD models select a constant value and employ it as a calibration parameter.

2 $$  B = \frac{\pi }{RT}\frac{\epsilon}{\tau \delta }\left[ {\left( {\frac{2}{3}\left( {\frac{8RT}{{\pi M_{w} }}} \right)^{\frac{1}{2}} r_{p}^{3} } \right)^{ - 1} + \left( {\frac{PD}{{p_{a} }}r_{p}^{2} } \right)^{ - 1} } \right]^{ - 1} $$

where R is the gas constant, $M_{w}$ is the water molecular weight and T is the membrane temperature. The membrane properties $r_{p}$, $\delta$, $\epsilon$ and $\tau$ are pore size, thickness, porosity and tortuosity, respectively. The membrane tortuosity and porosity can be calculated as^33^:

3 $$  \tau = \frac{1}{\epsilon} $$

$p_{a}$ is the air pressure inside the membrane pores and D is the water diffusion coefficient. The total pressure can be assumed constant in both DCMD’s channels module and equal to the atmospheric pressure. The viscous flow, as a result, would be negligible^34^. PD for the water–air mixture can be written:

4 $$ PD = 1.895 \times 10^{ - 5} T^{2.072} $$

where the water vapor pressure at each side of the membrane can be written:

5 $$ P_{w.p.m} \left( T \right) = P_{sat.w} \left( T \right) $$

here $P_{sat.w} \left( T \right)$ indicates the saturation pressure of water at the temperature T. The presence of salt at the feed channel causes the deviation of vapor from pure water. To considering impurities due to the NaCl, the activity coefficient ($\gamma_{w}$) can be determined as follows^35^:

6 $$ \gamma_{w} = 1 - 0.5X - 10X^{2} $$

The water vapor pressure at the feed channel can be expressed as:

7 $$ P_{w.f.m} \left( T \right) = \gamma_{w} \left( {1 - X} \right) P_{sat.w} \left( T \right) $$

here X is the molar solute concentration. The saturation pressure of water can be calculated by the Antoine equation. $P_{sat.w}$ is calculated as follows:

8 $$ P_{sat.w} \left( T \right) = {\exp}\left( {23.1964 - \frac{3816.44}{{T - 46.13}}} \right) $$

**Figure 2:**
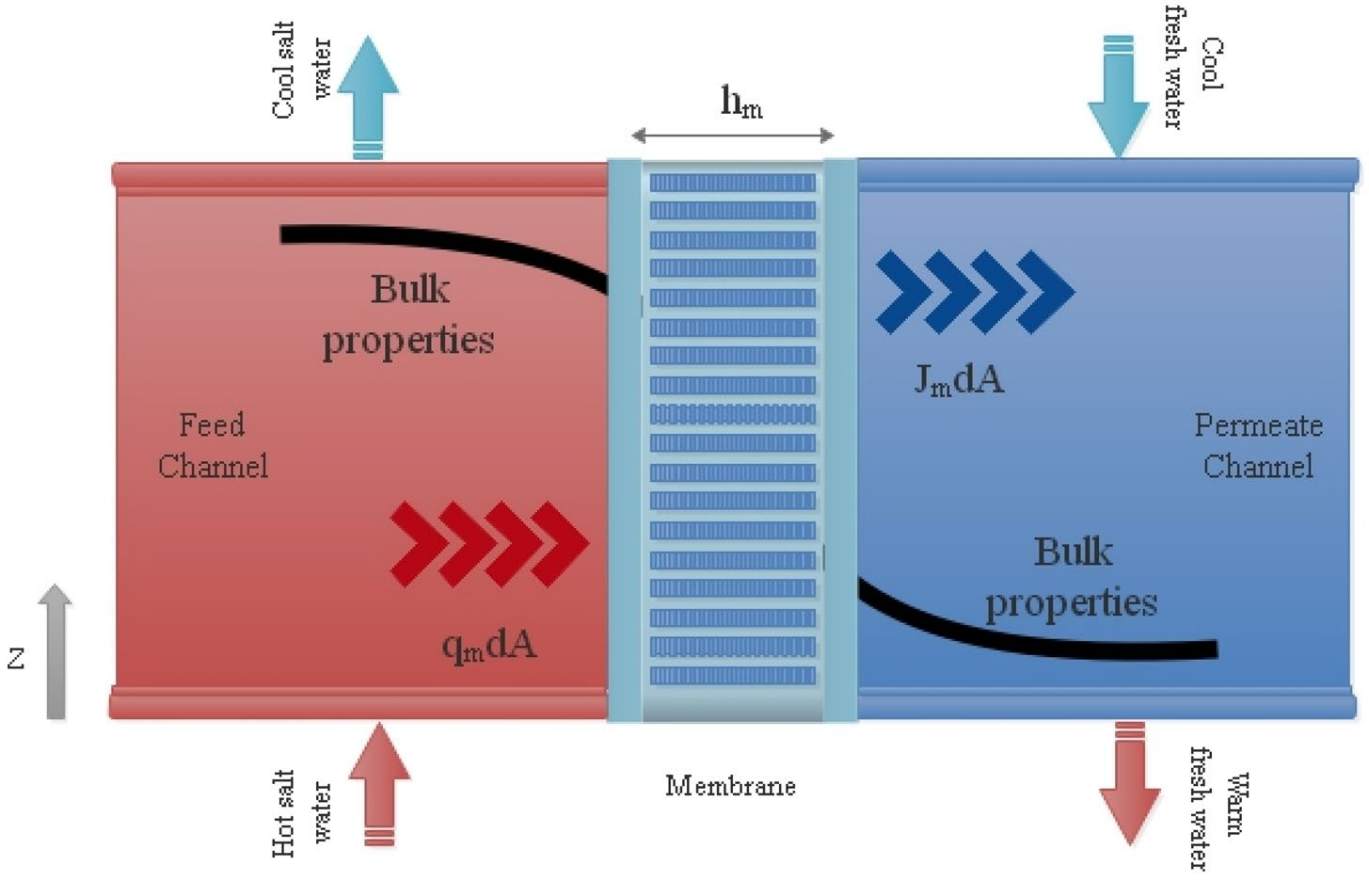
The schematic of control volumes.

Mass flux throughout the membrane is dependent on the temperature of channels. By applying the energy equation on each control volume, the temperature distribution along the channel can be obtained. Since the membrane module consists of three segments, the energy equation applies to each separately as is demonstrated in Fig. 2. In this regard, the energy equation is applied for the feed channel with the assumption of adiabatic walls:

9 $$ \dot{m}_{f} h_{f.b} |_{z + dz} = \dot{m}_{f} h_{f.b} |_{z} - \left( {J_{m} h_{v.f.m} + q_{m} } \right)dA $$

where $\dot{m}_{f}$ is the water mass flow rate, $h_{f.b}$ is the bulk enthalpy of liquid water and $h_{v.f.m}$ represents the vapor enthalpy at the membrane surface which flows through the membrane into the permeate channel. Also, $q_{m}$ is the heat flux through the membrane.

An energy balance for the permeate channel yields:

10 $$ \dot{m}_{p} h_{p.b} |_{z} = \dot{m}_{p} h_{p.b} |_{z + dz} + \left( {J_{m} h_{v.p.m} + q_{m} } \right)dA $$

where $\dot{m}_{p}$ is the water mass flow rate at the permeate channel. $h_{p.b}$ and $h_{v.p.m}$ represent the bulk liquid and vapor enthalpy at the permeate channel, respectively.

It is assumed that no mass is added or removed inside the membrane and all vapor flows from the feed channel into the permeate channel without any condensation in the membrane pores. Following equations, as a result, are given based on a continuity equation:

11 $$ \dot{m}_{f} |_{z + dz} = \dot{m}_{f} |_{z} - J_{m} dA $$

12 $$ \dot{m}_{p} |_{z} = \dot{m}_{p} |_{z + dz} + J_{m} dA $$

The vapor enthalpy at the boundary, $h_{v.m}$ can be considered as the latent heat of vaporization and saturated enthalpy of water at that temperature.

13 $$ h_{v.f.m} = h_{f.m} - h_{fg.f.m} $$

14 $$ h_{v.p.m} = h_{p.m} - h_{fg.p.m} $$

Substituting previous equations into Eqs. 9 and 10 yields:

15 $$ \dot{m}_{f} dh_{f.b} = - \left[ {J_{m} \left( {h_{fg.f.m} + h_{f.m} - h_{f.b} } \right) + q_{m} } \right]dA $$

16 $$ \dot{m}_{p} dh_{p.b} = - \left[ {J_{m} \left( {h_{fg.p.m} + h_{p.m} - h_{p.b} } \right) + q_{m} } \right]dA $$

here $dh_{f.b}$ and $dh_{p.b}$ represent the bulk enthalpy difference in the feed and permeate channel, respectively. The convective heat transfer of the membrane sides can be calculated as follows:

17 $$ J_{m} \left( {h_{fg.f.m} + h_{f.m} - h_{f.b} } \right) + q_{m} = h_{t.f} \left( {T_{f.b} - T_{f.m} } \right) $$

18 $$ J_{m} \left( {h_{fg.p.m} + h_{p.m} - h_{p.b} } \right) + q_{m} = h_{t.p} \left( {T_{p.m} - T_{p.b} } \right) $$

where $$T_{f.b}$$, $$T_{p.b}$$, $$T_{f.m}$$, $$T_{p.m}$$ are the bulk and membrane surface temperature of the feed and permeate channel, respectively. $$h_{t.f}$$ and $$h_{t.p}$$ are the convective heat transfer coefficient at the feed and permeate side which determined by the appropriate Nusselt number correlation. The total membrane thermal conductivity consists of two parts, the thermal conductivity of the solid part ($$k_{s}$$) and the heat conduction through the gas inside pores with the thermal conductivity of $$k_{g}$$. The thermal conductivity of air and water vapor, however, have the same order of magnitude so the thermal conductivity of water vapor is considered as $$k_{g}$$. By applying the parallel heat flow assumption through the gas inside pores and solid parts, the membrane thermal conductivity $$(k_{m} ) $$ can be calculated as follows ^36^:

19 $$  k_{m} =\epsilon k_{g} + \left( {1 -\epsilon } \right)k_{s} $$

The thermal conductivity of water vapor and PTFE membrane, which is employed in this study, are depended on the temperature. These values can be calculated using the following equations:

20 $$ k_{s} = 0.087 + 6.0 \times 10^{ - 4} T $$

21 $$ k_{v} = 2.72 \times 10^{ - 3} + 5.71 \times 10^{ - 5} T $$

Previous equations are valid between the temperature of 273–373 K. It is worth mentioning that the convection heat transfer within the membrane pores is negligible:

22 $$ q_{m} = k_{m} \frac{{T_{f.m} - T_{p.m} }}{{\delta_{m} }} $$

where $\epsilon$ and $\delta_{m}$ are porosity and membrane thickness, respectively.

For solving the above equations along the channel length, a proper Nusselt number is needed for each section of the channel. Various correlations based on the geometry and flow regime are suggested and used in the previous studies. At the present study, a general relationship for an arbitrary cross-sectional shape, considering the entry effects (both thermally and hydrodynamically), is applied^31^.

23 $$ Nu_{\sqrt A } \left( {z^{*} } \right) = \left[ {\left( {\frac{{f\left( {Pr} \right)}}{{\sqrt {z^{*} } }}} \right)^{m} + \left( {\left\{ {0.501\left( {\frac{{fRe_{\sqrt A } }}{{z^{*} }}} \right)^{\frac{1}{3}} } \right\}^{5} + \left\{ {3.86\left( {\frac{{fRe_{\sqrt A } }}{{8\sqrt \pi \varepsilon^{\gamma } }}} \right)} \right\}^{5} } \right)^{m/5} } \right]^{1/m} $$

Here $m$ is the blending parameter which is given by:

24 $$ {\text{m}} = {\text{a}} + {\text{b}} Pr^{1/3} $$

where a = 2.27 and b = 1.65. The product of friction factor (f) and Reynolds number which is defined based upon the square root of cross-sectional area, is given by:

25 $$ fRe_{\sqrt A } = \frac{12}{{\sqrt \varepsilon \left( {1 + \varepsilon } \right)\left[ {1 - \frac{192\varepsilon }{{\pi^{5} }}\tanh \left( {\frac{\pi }{2\varepsilon }} \right)} \right]}} $$

$f\left( {Pr} \right)$ for the uniform wall heat flux boundary condition is calculated as follows:

26 $$ f\left( {pr} \right) = \frac{0.886}{{\left[ {1 + \left( {1.909Pr^{1/6} } \right)^{9/2} } \right]^{2/9} }} $$

where $\varepsilon$, $z^{*}$, Pr and $\gamma$ are the aspect ratio of duct, dimensionless position of thermally developed flows, Prandtl number and shape parameter, respectively. $\gamma$ is considered − 0.3 due to the low aspect ratio of the duct. The input parameters of the DCMD is represented in Table 1.

**Table 1:** Geometrical and operational parameters of DCMD module.

	Value	Unit
**Geometrical parameters**		
Length, L	5	m
Width, w	0.5	m
Channel depth, $h_{ch}$	5	mm
Membrane thickness, $\delta_{m}$	200	μm
Porosity, $\epsilon$	0.7	–
Pore size, $r_{p}$	0.45	μm
**Operational parameters**		
Feed inlet temperature,$T_{in.f}$	70	°C
Permeate inlet temperature, $T_{in.p}$	25	°C
Feed inlet volumetric flow rate, $q_{in.f}$	10	l/min
Permeate inlet volumetric flow rate,$q_{in.f}$	10	l/min
Feed Inlet salinity, $s_{in}$	35,000	ppm

### Validation

**Figure 3:**
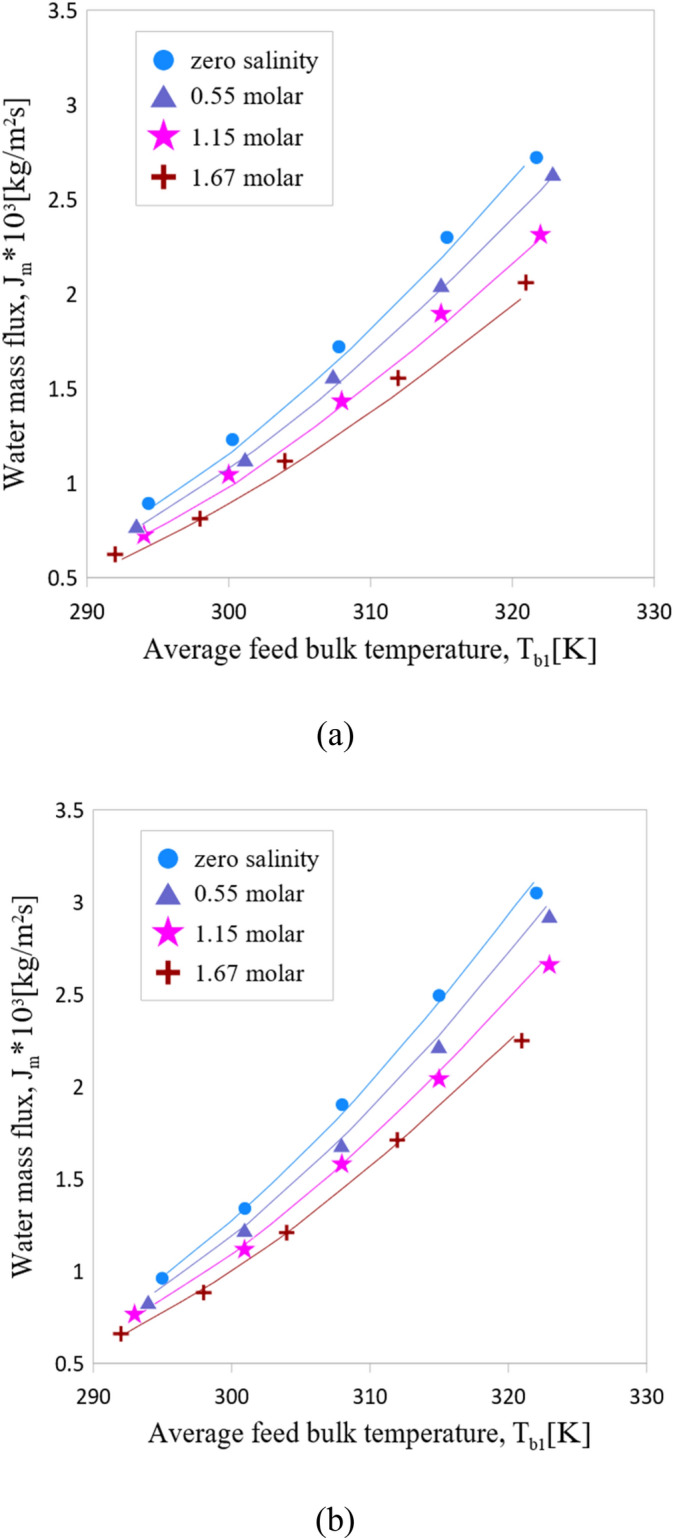
Water mass flux across membrane (**a**) Inlet flow rate, Q = 7 cm^3^/s (**b**) Inlet flow rate, Q = 11 cm^3^/s. Symbols indicate experimental data^25^ and lines show model results.

Based on the equations proposed in the modelling section, the DCMD model is developed and validated with the experimental data which are reported by Martínez-Díez and Vazquez-Gonzalez^37^. The PTFE membrane (with 80% void fraction, 60 μ$$\mathrm{m}$$ thickness, 0.2 mm nominal pore size) was implemented to perform experimental tests. The membrane module in that study was a counter flow flat sheet with 9 feed channels and 9 permeate channels. The water mass flux ($${J}_{m})$$ versus the average bulk temperature of the feed saltwater ($${T}_{b1}$$) are compared in Fig. 3 for two recirculation rates (7 and 11 cm^3^/s) at various salt feed water concentrations (0, 0.55, 1.15 and 1.67 molar NaCl). As is represented in Fig. 3 the simulation results are in an excellent agreement with experimental data. It is worth mentioning that $${T}_{b1}$$ in Fig. 3 is the average of the inlet and outlet bulk temperature of the feed channel.

### Constructal design and optimization

Speed-constrained Multi-objective particle swarm optimization (SMPSO) is an improved particle swarm optimization (PSO) characterized by the use of a strategy to limit the velocity of the particles. PSO is a bio-inspired evolutionary-based which imitating social behavior of bird flocking or fish schooling^38^. SMPSO use a strategy to put a constraint on particle velocity. In this regard, SMPSO produces new particles when the velocity becomes too high. Also, SMPSO benefits from polynomial mutation as a turbulence factor and external archive to store the non-dominated solutions found during the search^39^.

**Figure 4:**
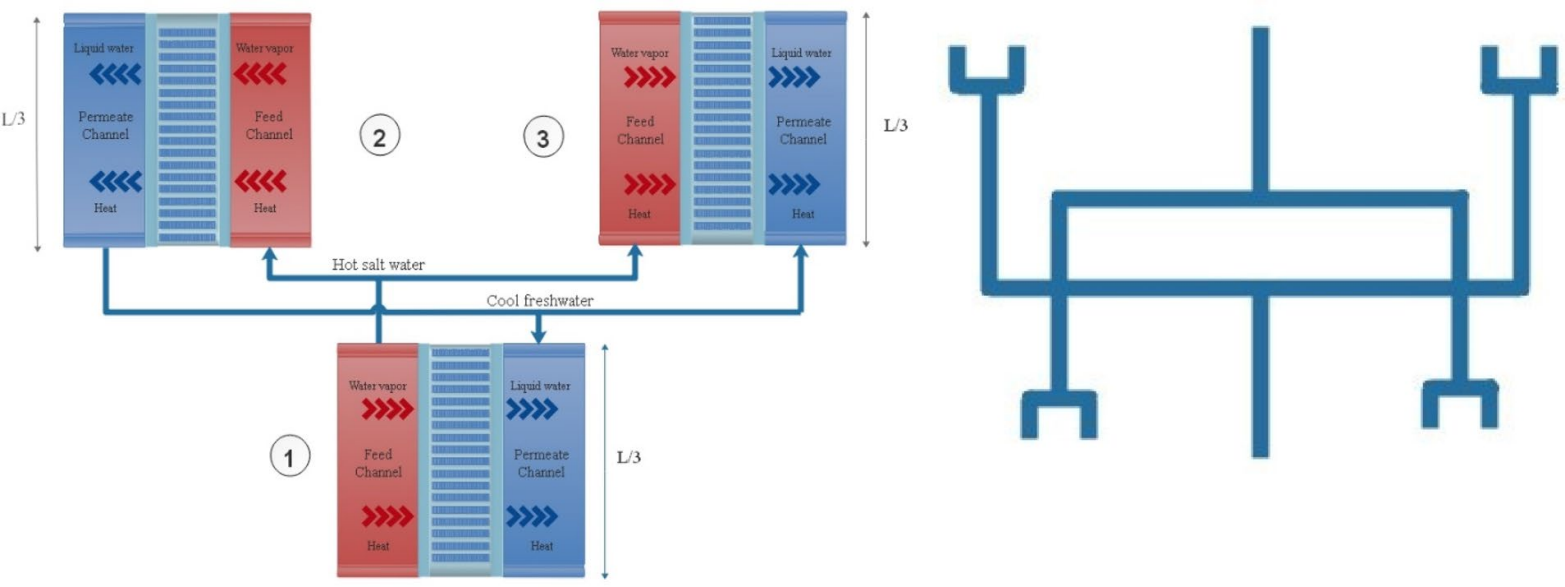
Schematic of constructal DCMD structure.

Constructal law, which was proposed for the first time in 1996^40^, states that for a flow system to persist in time it needs to evolve in a way that it provides easier access to its current^41^. The very epitome of this notion could be seen everywhere in nature^42^. Dendritic shapes, which is ubiquities in nature, are an icon of constructal design. These patterns could be seen in blood vessels, river basins and most recently the quantum footprint^43–45^. The present study tries to implement constructal law to enhance DCMD performance. While, the water mass flow rate and the size of channels are kept constant, as is demonstrated in Fig. 4, a new configuration is adopted to give the flow architecture the freedom to morph. The hot brine after entering in the feed channel is distributed into two other equally spaced feed channels (1 $\to$ 2,3). The same design is also considered for permeate channels. This configuration is also known as a canopy-to-canopy design.

Two different designs including constructal and conventional DCMD are considered for optimization. The schematic diagram of the constructal DCMD is illustrated in Fig. 4. The constructal DCMD consists of three parts. Each part of the constructal DCMD is one-third of the conventional DCMD. The inlet volume flow rate is divided into two equal portions while entering part 2 and 3. Nine design parameters including the inlet volume flow rate at the feed side, inlet volume flow rate at the permeate side, inlet feed water salinity, bulk temperature of the feed side, bulk temperature of the Permeate side, membrane length, membrane width, porosity and membrane pore size are considered for both conventional and constructal design. SMPSO algorithm is employed for optimization. The thermal efficiency and water mass flux are considered as two objective functions. The thermal efficiency of the DCMD can be defined as the ratio of latent heat of vaporization to the total heat transfer across the membrane which can written as:

27 $$ \eta = \frac{{J_{m} h_{fg} }}{{J_{m} h_{fg} + \frac{{k_{m} }}{\delta }\left( {T_{f.m} - T_{p.m} } \right)}} $$

The amount of the water mass flux ($J_{m}$) is calculated based on the previous equations. At the present study, the main goal of the optimization is to maximize both objective functions. The applicable ranges for relevant decision variables in this study are listed in Table 2.

**Table 2:** Ranges of decision variables in the present study.

Parameter	Symbol	Unit	Lower bound	Upper bound
Inlet volume flow rate at feed side	${q}_{r}$	l/s	0.1	0.6
Inlet volume flow rate at permeate side	${q}_{p}$	l/s	0.1	0.6
Inlet feed water salinity	${s}_{in}$	ppm	15,000	40,000
The bulk temperature of the feed side	$a$	°C	50	90
The bulk temperature of the Permeate side	$b$	°C	15	35
Membrane length	$L$	m	4	7
Membrane width	$W$	m	0.4	0.7
Porosity	$kisi.\varepsilon$	–	0.4	0.9
Membrane pore size	${r}_{p}$	m	0.4e−6	0.9e−6

## Results and discussion

**Figure 5:**
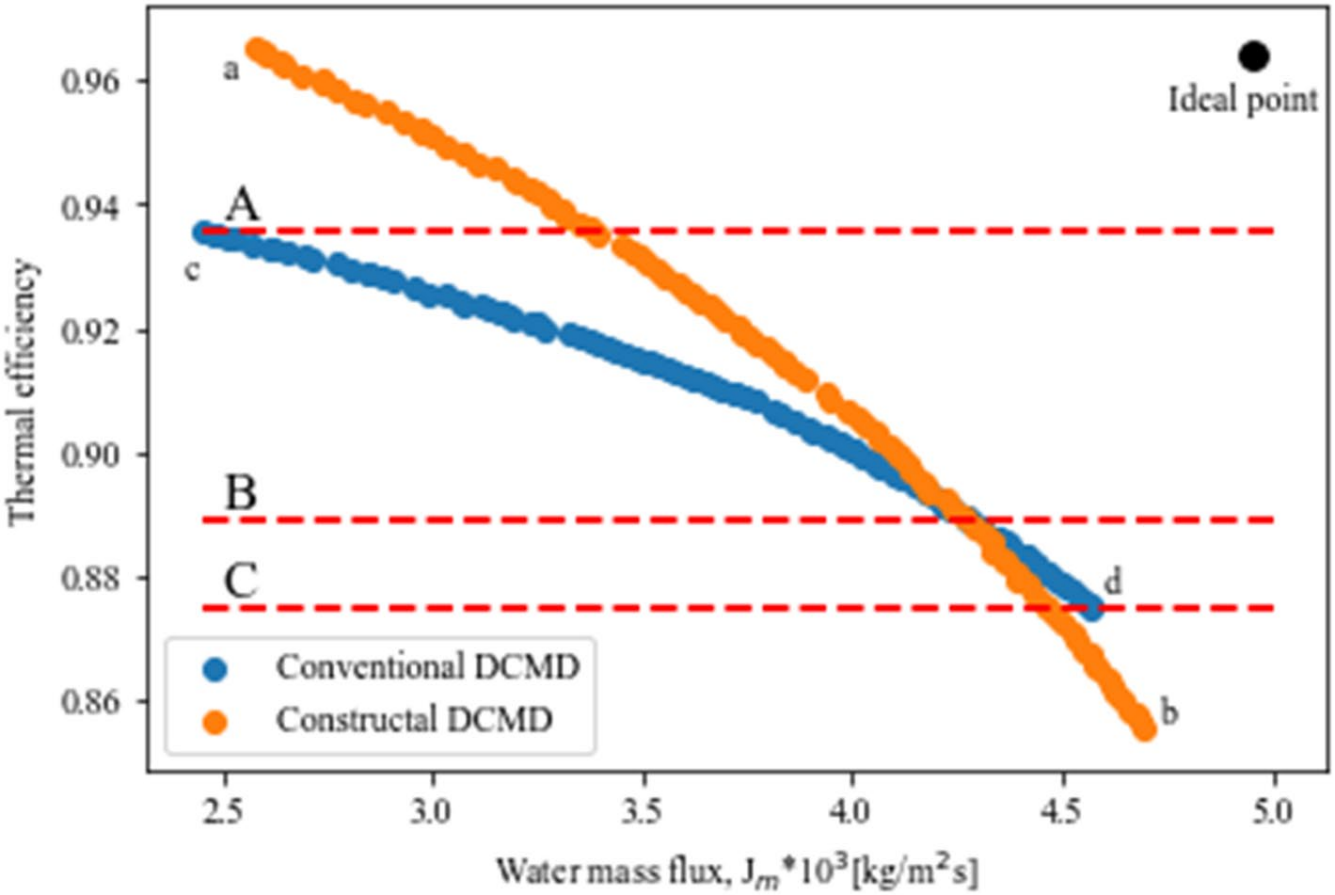
Pareto frontiers for conventional and constructal DCMD designs.

The Pareto frontier of the conventional and constructal designs is represented in Fig. 5. As is shown the constructal design indicates a better performance before the water mass flux of about 4.4 kg/m^2^s as it is closer to the ideal point. Its performance, however, drops after the efficiency of 89% and mass flux of 4.4 kg/m^2^s. After this point the conventional design has a better performance. Therefore, for the efficiencies below 89% or mass fluxes more than 4.4 kg/m^2^s, the conventional design is preferable to the constructal design. The equilibrium point is closer to the constructal Pareto frontier so as a result, the constructal design can strike a better balance between the thermal efficiency and water mass flux. The Pareto optimal front of the constructal DCMD design is started from point (a), showing the single objective optimization (Thermal efficiency). Point (a) has the highest thermal efficiency. Its water mass flux, however, is at its lowest value. The constructal DCMD design has the thermal efficiency of 96.5% at point (a) meanwhile, its mass flux is 2.57 kg/m^2^s. On the contrary, the constructal DCMD has the largest mass flux with the amount of 4.69 kg/m^2^s and the thermal efficiency of 85.5% at point (b). Point (c) indicates the single-objective optimization (thermal efficiency) of the conventional DCMD which has the value of 93.5%. Point (b) is the most suitable design from a water mass flux viewpoint as it has the largest quantity (4.57 kg/m^2^s) among other points. Nevertheless, it is the worst design for the thermal efficiency. Aforementioned points are the result of single objective optimizations. As is illustrated, the conventional design is dominated by the constructal design from a thermal efficiency perspective, in contrast, the conventional design can have a larger amount of mass flux as point (b) is lower than the point (d). Three lines (A, B and C) are added to assess the impact of the constructal design on DCMD performance. Line A shows that with the same thermal efficiency of 93.5%, the constructal design can improve the water mass flux by 37.5% from 2.45 to 3.37 kg/m^2^s which is a considerable enhancement. Line C, on the other hand, indicates that with the same thermal efficiency of 87.5%, the constructal design leads to decreasing the mass flux by 2.4%. Line B is considered as the reference line inasmuch as the constructal design is more appropriate above this line. In contrast, the conventional DCMD is more suitable below this line.

**Figure 6:**
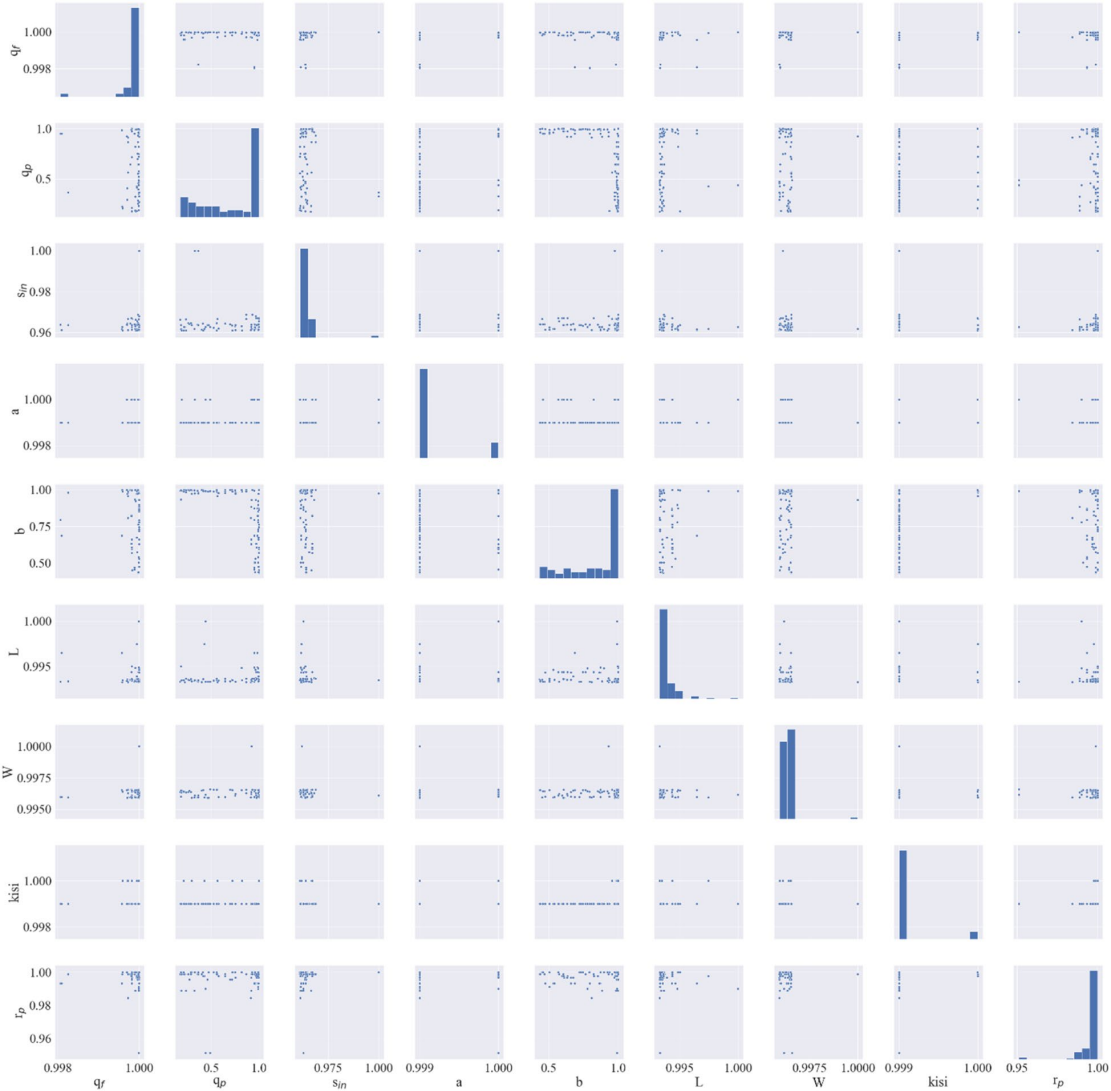
Pair plots of decision variables.

Since in the most parts the constructal design has a superior, the design and operational parameters of this design will be discussed. The correlation between each parameter (Pair Plot) is illustrated in Fig. 6. Pair plot illustrates the relation between decision variables, helping to identify the influence of parameters on each other. The diagonal of the pair plot shows the distribution of design parameters. Description of each decision variable is shown in Table 2. As is represented in Fig. 6 most of the design and operational parameters follow a dominated trend which can provide a useful insight into what range of decision variables can lead to an optimal design. Decision-makers and designers, as a result, can use these design parameters to design an optimal constructal DCMD. It can also suggest optimum operational conditions for the constructal DCMD.

**Figure 7:**
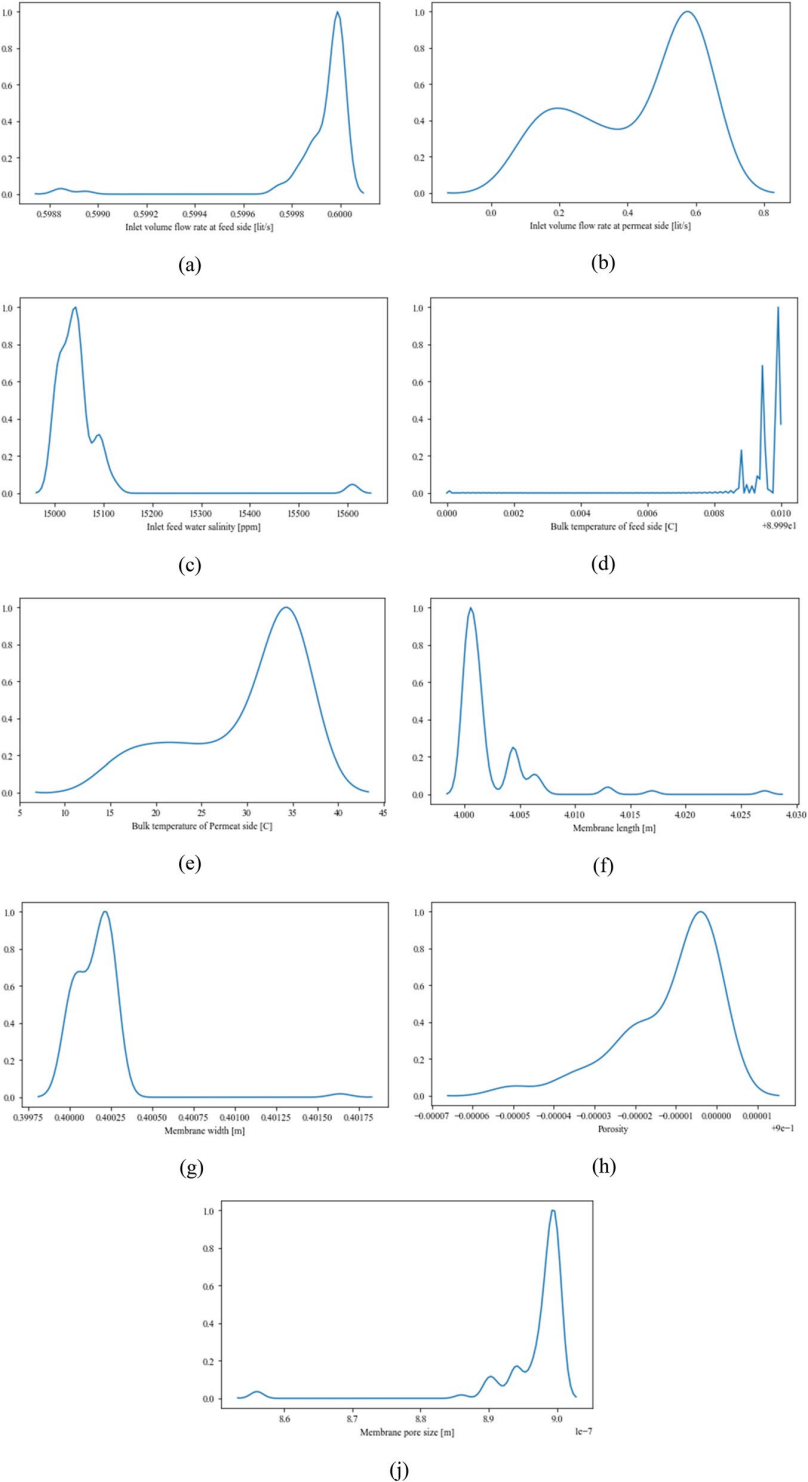
Frequency distribution of decision variables.

For precise investigation, the distribution of each decision variable is represented in Fig. 7.

The frequency distribution of decision variables reveals the most appropriate value for each decision variable. Generally, the frequency distribution of decision variables can be categorized into two main sections. First, design and operational variables that have one peak (Fig. 7a, h). In such categories, there is a dominant value that causes an optimal constructal DCMD design. On the other hand, some frequency distributions of the decision variables have two peaks. In such cases, each optimal design could have one of these values. The very epitome of this could be seen in Fig. 7b. Also, there is a possibility that a decision variable has three peaks (Fig. 7d). As is shown in Fig. 7a, almost all of the optimal points have the value of 0.59 l/s which means it is the most suitable quantity for the inlet volume flow rate at the feed side. Figure 7b shows the optimal points for the inlet volume flow rate at the permeate side. As is discussed before, it has two peaks (0.2 and 0.6 l/s). Choosing one of these values are depended on other decision variables. Figure 7c represents the most suitable inlet feed water salinity. This figure reveals that the best inlet feed water salinity for the constructal DCMD is between 15,000 and 15,100 ppm. In other words, the constructal DCMD will be performed optimally if it works in this range of inlet feed water salinity. Figure 7d shows the most optimal bulk temperature. Although each peak could lead to an optimal operation condition, the highest one (89 °C) is the most dominant value. The most frequent value of the permeate side bulk temperature, however, is 34 °C as is shown in Fig. 7e. Most of the appropriate values for the membrane length, membrane width, porosity and membrane pore size, with the same procedure, are 4 m, 0.4 m, 0.89 and 0.89 e−7, respectively.

**Figure 8:**
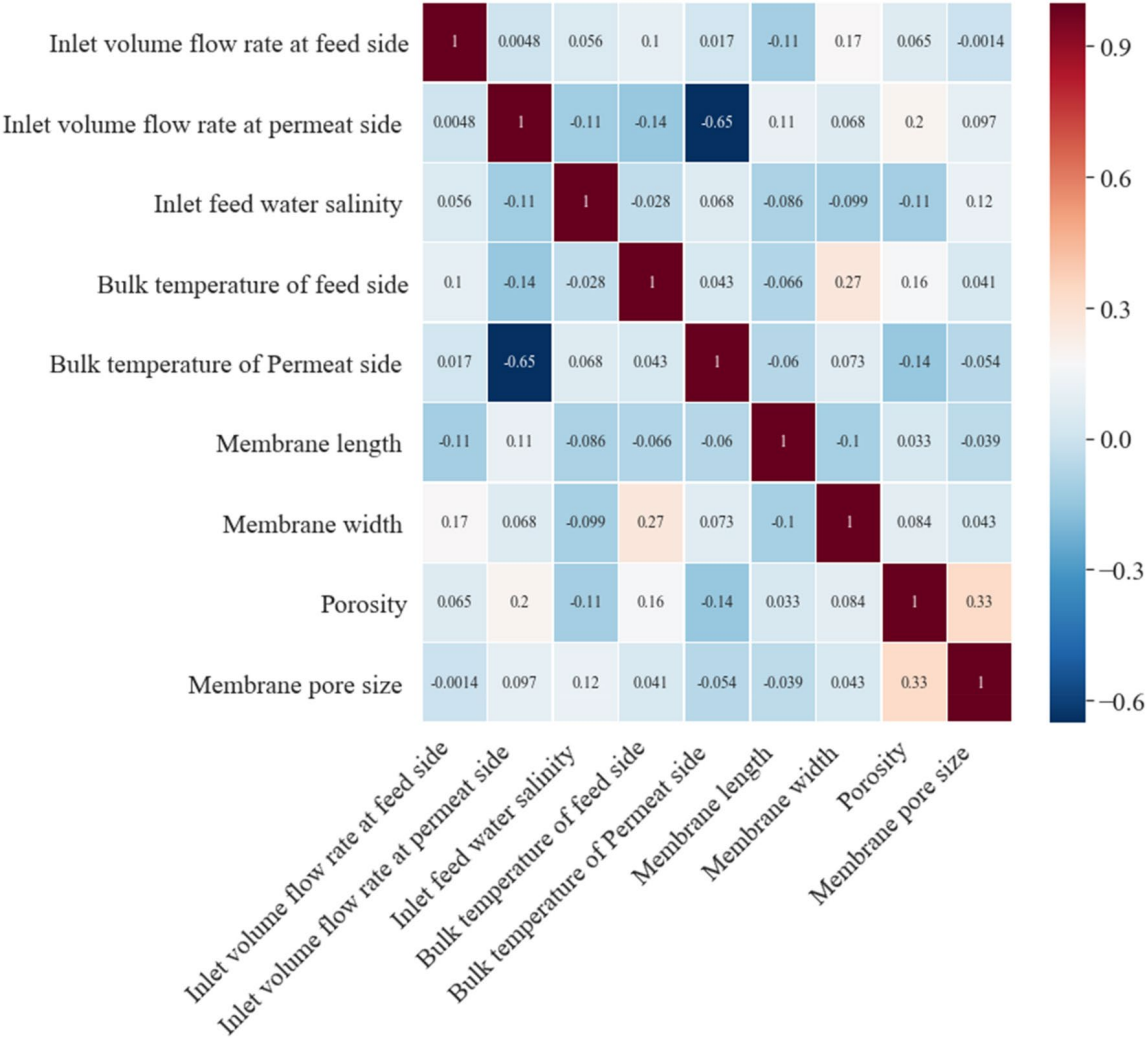
The Pearson correlation coefficient of decision variables.

Another important question is whether decision variables have a correlation with each other or not. Figure 8 shows the Pearson correlation coefficients to determine whether there is any relation between decision variables. This can help designers to understand what effect a change in one parameter has on the other parameters so they can modify design parameters effectively.

Pearson’s correlation coefficient calculated by dividing the covariance of the variables, which are decision variables here, to the product of their standard deviations. As is shown there is some conspicuous relation between some design parameters which at the first glance seems to have nothing to do with each other. Starting from the inlet volume flow rate and bulk temperature at the permeate side. As is demonstrated in Fig. 8 these variables move in the opposite direction. In broad terms, for having an optimized constructal DCMD, if the inlet volume flow rate at the permeate side is increased, the bulk temperature of the permeate side must be decreased to keep the constructal DCMD optimized. Another interesting correlation exists between the porosity and membrane pore size. These two design parameters are move in the same direction so for having an optimized constructal DCMD, the quantity of both variables have to increase or decrease simultaneously. The membrane width and bulk temperature of the feed side have a weaker yet very interesting relation. As is shown these parameters move in the same direction. More correlations can be discovered as is shown in Fig. 6. Pearson’s correlation coefficient more than 0.3 or less than − 0.3 can be called as a moderate or strong correlation between parameters and can have a conspicuous impact on objectives. These relations can help decision-makers to increase or decrease the values of design parameters more wisely to keep the constructal DCMD optimized.

**Figure 9:**
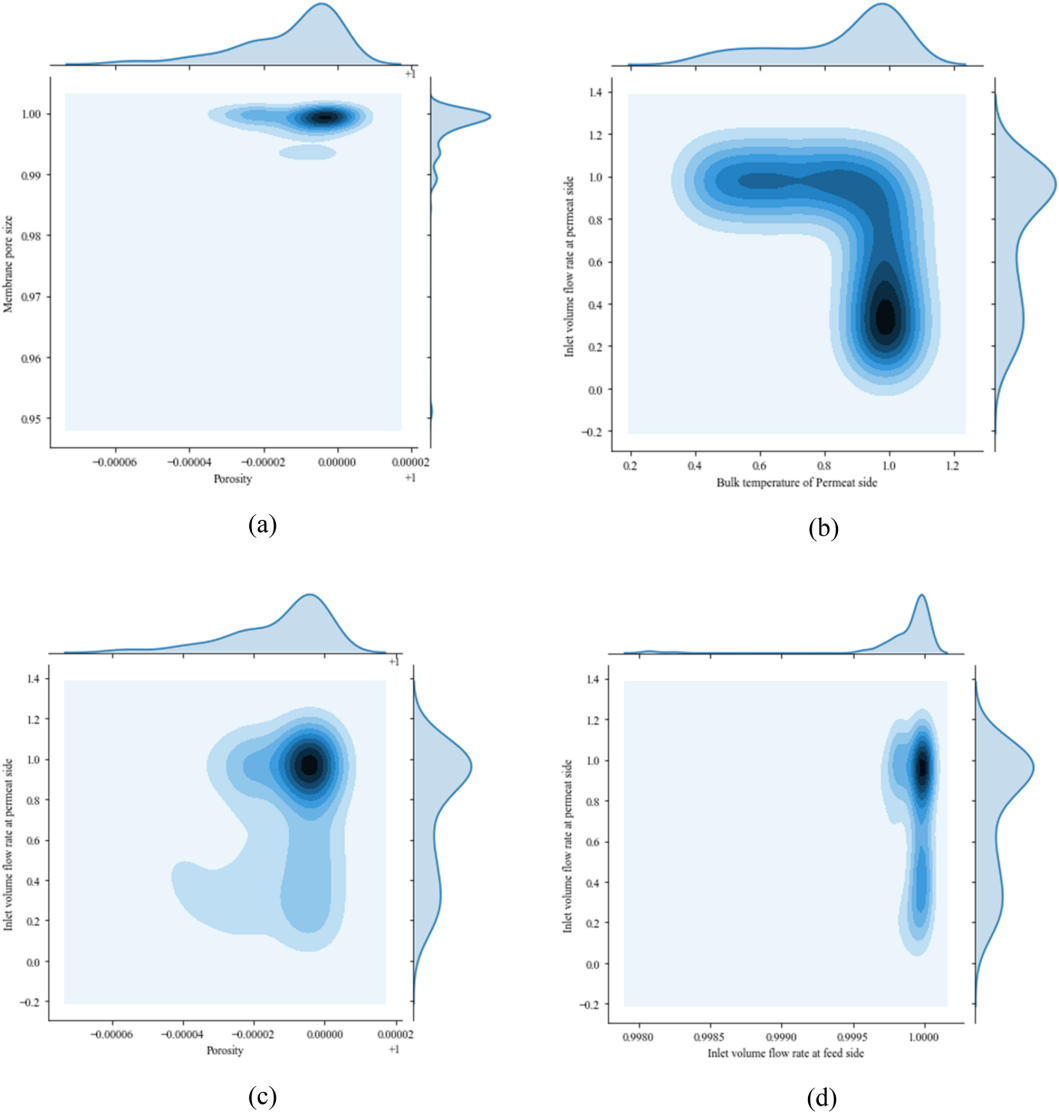
Marginal plot of decision variables.

The marginal plot of several decision variables is also represented in Fig. 9. Marginal plots help to examine decision variables distributions and how designers should choose decision variables to build an optimal constructal DCMD. Decision variables which have a considerable Pearson’s correlation coefficient are shown in Fig. 9. For easier visualization data have been normalized between 0 and 1. Figure 9a shows that both the membrane pore size and porosity have to be chosen from their highest frequency as the deepest part of the Fig. 9a is located in this part. Figure 9b illustrates that the most optimal points happen when the bulk temperature is chosen from its high frequency values while the inlet volume flow rate at the permeate side should choose from its second peak. Other values of design parameters can also be employed by designers based on other parts of the figures but the most optimal designs happen with the aforementioned values. Figure 9c, with the same procedure, reveals that the porosity and membrane pore size have to be selected from their highest frequency value. Figure 9d can be analyzed with the same method.

## Conclusion

At the present study, a novel DCMD design is proposed based on the constructal theory. Flow and membrane properties are estimated by the temperature-dependent correlations to reduce the model input parameters. A generalized Nusselt number correlation is employed for the thermal and hydrodynamic flow. The average mass flux which is calculated by this model is in a very good agreement with experimental data. SMPSO optimization algorithm is implemented to assess whether the constructal design can improve the performance of the DCMD. Results reveal that the constructal DCMD design leads to better performance under the water mass flux of about 4.4 kg/m^2^s or less than that. The conventional design, on the other hand, illustrates better performance after this point. Also, the results indicate that the constructal design is more desirable for higher thermal efficiencies. The constructal design dominates the conventional design from a thermal efficiency perspective, in contrast, the conventional design can perform under higher mass flux. For instance, the optimization outcomes show that with the same thermal efficiency of 93.5% the constructal design can improve the water mass flux by 37.5% from 2.45 to 3.37 kg/m^2^s. In the next step, the pair plot is employed to investigate the relation between decision parameters. It indicates that there are dominated trends among design and operational parameters which can provide a useful insight into what range of decision variables can lead to an optimal design. The frequency distribution of decision variables demonstrates that decision variables could be categorized into two main parts. First, operational and design variables that have one peak which means that there is one dominant value that causes an optimal constructal DCMD design. Second, decision variables that have two or three peaks. In such categories, each peak could lead to optimal design. The quantities of each parameter are discussed in the results and discussion section. The results show that the bulk temperature and inlet volume flow rate of the permeate side move in the opposite direction which means for obtaining an optimized constructal DCMD, if the bulk temperature of the permeate side is increased, the inlet volume flow rate at the permeate side must be decreased to keep the constructal DCMD optimized. Also, results demonstrate that the amount of the membrane pore size and porosity must increase or decrease simultaneously. Data disclose that the membrane pore size and porosity have to be chosen from their highest frequency.
